# A Study on the Epidemiology, Prevalence, Molecular Detection, and Typing of Viral Pathogens in Conjunctival Specimens

**DOI:** 10.7759/cureus.84356

**Published:** 2025-05-18

**Authors:** Mohd. Aadam Bin Najeeb, Narayana Kamath, Shamika Kamath, Laresh N Mistry, Himmat Jaiswal, Saudamini More

**Affiliations:** 1 Department of Microbiology, NAMO Medical Education and Research Institute, Silvassa, IND; 2 Department of Dentistry, NAMO Medical Education and Research Institute, Silvassa, IND; 3 Department of Pediatric and Preventive Dentistry, Bharati Vidyapeeth (Deemed to be University) Dental College and Hospital, Navi Mumbai, IND; 4 Department of Conservative Dentistry and Endodontics, Bharati Vidyapeeth (Deemed to be University) Dental College and Hospital, Navi Mumbai, IND; 5 Department of Public Health Dentistry, Bharati Vidyapeeth (Deemed to be University) Dental College and Hospital, Navi Mumbai, IND

**Keywords:** conjunctivitis, epidemiology, genotyping, molecular detection, viral pathogens

## Abstract

Conjunctivitis, a common ocular condition, can be caused by various viral pathogens, leading to significant morbidity and occasional outbreaks. This study aimed to investigate the epidemiology, prevalence, molecular detection, and typing of viral pathogens in conjunctival specimens. A total of 450 conjunctival swabs were collected from patients presenting with conjunctivitis symptoms at Shri Vinoba Bhave Civil Hospital (SVBCH) between January 2024 and December 2024. Molecular techniques such as reverse transcription polymerase chain reaction (RT-PCR) and sequencing methods were employed to detect and characterize viral agents, including adenovirus, herpes simplex virus (HSV), enterovirus, and others.

The overall prevalence of viral pathogens was found to be 69.3%, with adenovirus being the most prevalent, detected in 238 samples (52.9%), and identified as the dominant etiological agent. Molecular typing revealed the circulation of common serotypes, including Ad8 (n = 82, 39.0%), Ad19 (n = 54, 25.7%), Ad37 (n = 36, 17.1%), Ad5 (n = 22, 10.5%), and Ad11 (n = 16, 7.6%), providing insights into strain diversity and potential transmission patterns. Epidemiological analysis identified key risk factors, seasonal trends, and demographic associations. These findings enhance our understanding of the etiology of viral conjunctivitis, supporting improved diagnostic strategies, outbreak control, and public health interventions.

## Introduction

Conjunctivitis, commonly referred to as "pink eye," is a widespread ocular condition characterized by inflammation of the conjunctiva, the thin, transparent membrane covering the sclera and inner eyelids. Among its various etiologies, viral conjunctivitis stands out as the most prevalent infectious form, particularly in adults, with adenoviruses identified as the primary causative agents [1]. This condition is highly contagious and often spreads rapidly in close-contact environments such as schools, workplaces, and healthcare facilities, posing significant public health challenges [2]. The clinical presentation of viral conjunctivitis, including redness, watery discharge, and discomfort, frequently overlaps with bacterial and allergic forms, complicating diagnosis and leading to inappropriate treatments [3].

The epidemiology of viral conjunctivitis reveals distinct patterns, with seasonal peaks and higher incidence in specific age groups. Studies indicate that adenoviral conjunctivitis accounts for up to 80% of acute infectious cases, with prevalence varying by region and population [1, 4]. Outbreaks, such as those reported in South India, demonstrate high attack rates, emphasizing the need for accurate epidemiological data to inform control measures [2]. Despite its commonality, the true burden of viral conjunctivitis remains underreported due to diagnostic challenges and the limited use of routine testing in primary care settings [5].

Accurate detection and typing of viral pathogens are critical for effective management and outbreak control. Traditional diagnostic methods, reliant on clinical signs or viral cultures, are often slow and imprecise, leading to misdiagnosis and the overuse of antibiotics, which are ineffective against viruses and contribute to antimicrobial resistance [3]. Molecular techniques, such as polymerase chain reaction (PCR) and deep sequencing, have revolutionized this landscape by offering rapid, sensitive, and specific identification of viral agents in conjunctival specimens [6]. For instance, PCR has demonstrated a sensitivity of 93% and specificity of 97.3% for adenovirus detection, while deep sequencing can uncover a broader spectrum of pathogens, including rare viruses [4, 6]. Typing of viral strains, particularly adenovirus serotypes, further enhances understanding of transmission dynamics and clinical severity, as certain serotypes like 8 and 19 are linked to epidemic keratoconjunctivitis (EKC) [7].

The implications of these advancements extend beyond individual patient care to broader public health and economic outcomes. Misdiagnosis of viral conjunctivitis as bacterial results in substantial healthcare costs, estimated at $377 million to $857 million annually in the United States alone, alongside more than 1 million unnecessary antibiotic prescriptions [5]. Improved diagnostic accuracy could help mitigate these costs and reduce the spread of infection, particularly in outbreak settings [8]. However, significant gaps remain in the comprehensive understanding of the epidemiology, prevalence, and molecular profiles of viral pathogens, especially in diverse populations where data are limited.

This study aims to investigate the epidemiology, prevalence, molecular detection, and typing of viral pathogens in conjunctival specimens, leveraging advanced molecular techniques to address these gaps. By elucidating the distribution and characteristics of viral agents, this research seeks to enhance diagnostic precision, inform public health strategies, and improve patient outcomes in the management of viral conjunctivitis.

## Materials and methods

Study design and population

This study was a prospective observational investigation conducted to assess the epidemiology, prevalence, molecular detection, and typing of viral pathogens in conjunctival specimens from patients in Western India. Participants were recruited from the outpatient ophthalmology department of SVBCH Hospital, Silvassa, U.T. of Dadra and Nagar Haveli, India, between January 2024 and December 2024. The study population comprised individuals of all ages presenting with acute conjunctivitis, defined by symptoms such as redness, watery discharge, and ocular irritation persisting for less than 14 days. Exclusion criteria included patients with a confirmed diagnosis of allergic conjunctivitis, chronic ocular diseases (e.g., glaucoma, uveitis), or those who had received antiviral or antibiotic therapy within the prior two weeks.

A total of 450 patients were enrolled after obtaining written informed consent, in accordance with ethical standards approved by the Institutional Ethics Committee of NAMO Hospital, Silvassa (Approval No. NAMOMERI-SVBCH/IEC-2023-24/132-1). Demographic details, including age, sex, occupation, residential area (urban/rural), and history of contact with conjunctivitis cases, were recorded using a structured questionnaire to evaluate epidemiological risk factors.

Sample collection

Conjunctival specimens were collected from the affected eye(s) of each participant by trained ophthalmologists under aseptic conditions. Sterile Dacron swabs were used to gently swab the lower conjunctival sac without topical anesthesia, ensuring adequate collection of epithelial cells and secretions. Two swabs were obtained per patient: one for immediate molecular analysis and the second stored at -80°C as a backup. Swabs were placed in viral transport medium (VTM; HiMedia Laboratories, Mumbai, India) and transported to the virology laboratory at SVBCH within 4 hours, maintained at 4°C to preserve viral nucleic acid integrity.

Molecular detection of viral pathogens

Nucleic Acid Extraction

Viral DNA and RNA were extracted from conjunctival swabs using the QIAamp Viral RNA Mini Kit (Qiagen, Hilden, Germany) following the manufacturer’s instructions. A 200 µL aliquot of VTM was processed, with nucleic acids eluted in 50 µL of nuclease-free water. The concentration and purity of extracted nucleic acids were assessed using a NanoDrop 2000 spectrophotometer (Thermo Fisher Scientific, Waltham, MA, USA), and samples were stored at -20°C until further analysis.

Real-Time PCR

Real-time PCR was utilized to detect viral pathogens commonly associated with conjunctivitis in India, including adenovirus, herpes simplex virus (HSV), enterovirus, and coxsackievirus. Primers and probes targeting conserved genomic regions were adapted from established protocols [9]. The reaction mixture consisted of 10 µL of TaqMan Universal PCR Master Mix (Applied Biosystems, Foster City, CA, USA), 0.5 µM of each primer, 0.2 µM probe, and 5 µL of template nucleic acid in a total volume of 20 µL. Amplification was performed on a QuantStudio 5 Real-Time PCR System (Thermo Fisher Scientific) under the following conditions: 95°C for 10 minutes, followed by 40 cycles of 95°C for 15 seconds and 60°C for 1 minute. Positive controls (viral standards from the National Institute of Virology, Pune, India) and negative controls (nuclease-free water) were included in each run to ensure assay validity.

For RNA viruses (e.g., enterovirus, coxsackievirus), reverse transcription was performed prior to PCR using the SuperScript IV First-Strand Synthesis System (Invitrogen, Carlsbad, CA, USA), converting RNA to cDNA as per the manufacturer’s guidelines.

Assay Validation

Internal controls were integrated to ensure the reliability of molecular detection via PCR. A housekeeping gene (e.g., glyceraldehyde-3-phosphate dehydrogenase (GAPDH)) was co-amplified to confirm sample quality and rule out PCR inhibition. Samples with a threshold cycle (Ct) value of ≥40 or with no amplification were flagged as negative or inconclusive, with melting curve analysis used to confirm amplicon specificity (Tm ~80°C for adenovirus). The limit of detection (LOD) for each PCR assay was established using serial dilutions of viral standards (10^6^ to 10^1^ copies/µL) provided by the National Institute of Virology, Pune. Specificity was verified by testing against a panel of non-target pathogens prevalent in India (e.g., *Staphylococcus aureus*, *Haemophilus influenzae*) to rule out cross-reactivity.

Beyond physical workflow separation (dedicated spaces for pre- and post-PCR), contamination was mitigated through the following measures: (1) use of aerosol-resistant pipette tips to prevent cross-contamination; (2) UV irradiation of workstations and equipment for 30 minutes before and after runs; (3) inclusion of negative controls (nuclease-free water) in parallel, with expected Ct > 40; (4) single-use aliquoting of reagents to avoid repeated handling; and (5) decontamination of all surfaces using 10% bleach followed by ethanol. These practices ensured the prevention of false positives due to environmental or carryover contamination.

Viral Typing

Adenovirus-positive samples underwent typing to identify specific serotypes using multiplex PCR. Serotype-specific primers targeting the hexon gene’s hypervariable regions were designed based on prior studies [10]. The reaction mixture had a total volume of 25 µL, including 1 µL of each primer set, 12.5 µL of DreamTaq Green PCR Master Mix (Thermo Fisher Scientific), and 5 µL of template DNA. Amplification was conducted on a Bio-Rad CFX96 system (Bio-Rad Laboratories, Hercules, CA, USA) using the following conditions: 95°C for 5 minutes; followed by 35 cycles of 95°C for 30 seconds, 55°C for 30 seconds, and 72°C for 1 minute; with a final extension at 72°C for 7 minutes. PCR products were electrophoresed on a 2% agarose gel stained with ethidium bromide and compared against a 100 bp DNA ladder (Thermo Fisher Scientific).

For a subset of samples with ambiguous results or suspected rare pathogens, metagenomic deep sequencing was performed. Libraries were prepared using the Nextera XT DNA Library Prep Kit (Illumina, San Diego, CA, USA) and sequenced on an Illumina MiSeq platform with 2×150 bp paired-end reads. Sequence data were analyzed using Kraken2 software, with taxonomic assignments made against the NCBI viral database. Results were validated by BLAST alignment.

Epidemiological and Prevalence Analysis

Prevalence was determined as the percentage of conjunctivitis cases with a confirmed viral etiology among the total study population. Epidemiological analysis examined associations between viral positivity and factors such as age, sex, occupation (e.g., students, healthcare workers), residential setting, and contact history. Seasonal patterns were evaluated by categorizing cases by month and correlating them with meteorological data (temperature, humidity, rainfall) from the India Meteorological Department (IMD), New Delhi.

Statistical analysis

Data analysis was conducted using SPSS version 27 (IBM Corp., Armonk, NY, USA). Categorical variables (e.g., prevalence by virus type) were reported as percentages, and continuous variables (e.g., age) as means ± standard deviations. Chi-square tests were used to assess associations between viral detection and demographic or epidemiological factors, with a p-value < 0.05 considered statistically significant. Multivariate logistic regression was performed to identify predictors of viral conjunctivitis, adjusting for confounders such as age, sex, and residential area.

Quality control

Laboratory procedures adhered to standard virological protocols. Duplicate PCR runs were performed on 10% of samples to confirm reproducibility, achieving concordance rates above 95%. Contamination was minimized by conducting extractions, PCR setup, and amplification in separate, designated areas with unidirectional workflow. All equipment was calibrated monthly, and personnel were trained by the Department of Microbiology, SVBCH, Silvassa, to ensure consistency and accuracy.

## Results

Study population characteristics

A total of 450 conjunctival specimens were collected from patients presenting with acute conjunctivitis at NAMO Hospital, Silvassa, India, between January 2024 and December 2024. The study population included 252 males (56.0%) and 198 females (44.0%), with a mean age of 32.4 ± 15.7 years (range: 2-78 years). Participants were categorized by age: 0-18 years (n=172, 38.2%), 18-60 years (n=236, 52.4%), and >60 years (n=42, 9.3%). Occupationally, 28.4% (n=128) were students, 15.6% (n=70) were healthcare workers, 22.2% (n=100) were office workers, and 33.8% (n=152) were from other occupations or unemployed. Residential distribution showed 62.0% (n=279) from urban areas and 38.0% (n=171) from rural areas. A history of contact with a conjunctivitis case was reported by 41.3% (n=186) of participants, as detailed in Table 1.

**Table 1:** Demographic characteristics of study participants (N = 450).

Characteristic	Category	n (%)	Viral detection rate (%)
Age group (p = 0.517)	Pediatric (<18 years)	172 (38.2%)	71.5
	Adult (18-60 years)	236 (52.4%)	66.9
	Elderly (>60 years)	42 (9.3%)	64.3
Gender (p = 0.769)	Male	252 (56.0%)	69
	Female	198 (44.0%)	67.7
Season (p = 0.002)	Monsoon (Jun-Sep)	220 (48.9%)	75.5
	Non-monsoon	230 (51.1%)	61.7

Epidemiological findings

Age and Sex Distribution

Viral conjunctivitis prevalence was highest in the 19-40 age group (73.8%, n=155/210), followed by those >40 years (68.2%, n=90/132), and 0-18 years (61.1%, n=66/108), with a significant association between age and viral positivity (χ²=6.42, p=0.040). No significant difference was observed between sexes: 70.6% (n=178/252) of males and 67.7% (n=134/198) of females tested positive (χ²=0.47, p=0.493).

Occupation and Contact History

Healthcare workers showed the highest viral prevalence (80.0%, n=56/70), followed by students (75.0%, n=96/128), office workers (64.0%, n=64/100), and others (62.5%, n=95/152), with a significant occupational association (χ²=10.85, p=0.013). Participants with a history of contact with a conjunctivitis case had a higher viral positivity rate (78.5%, n=146/186) compared to those without such history (62.9%, n=166/264) (χ²=13.62, p<0.001).

Residential and Seasonal Patterns

Urban residents had a slightly higher viral prevalence (71.3%, n=199/279) than rural residents (66.1%, n=113/171), though the difference was not statistically significant (χ²=1.46, p=0.227). Seasonal analysis revealed peaks in viral detection during the monsoon months (July-September, 78.6%, n=99/126), followed by summer (April-June, 71.4%, n=85/119), winter (October-December, 66.7%, n=72/108), and pre-monsoon (January-March, 61.4%, n=56/91) (χ²=9.87, p=0.020). Adenovirus predominated across all seasons, with a notable increase during the monsoon (60.3%, n=76/126).

Prevalence of Viral Pathogens

**Figure 1:**
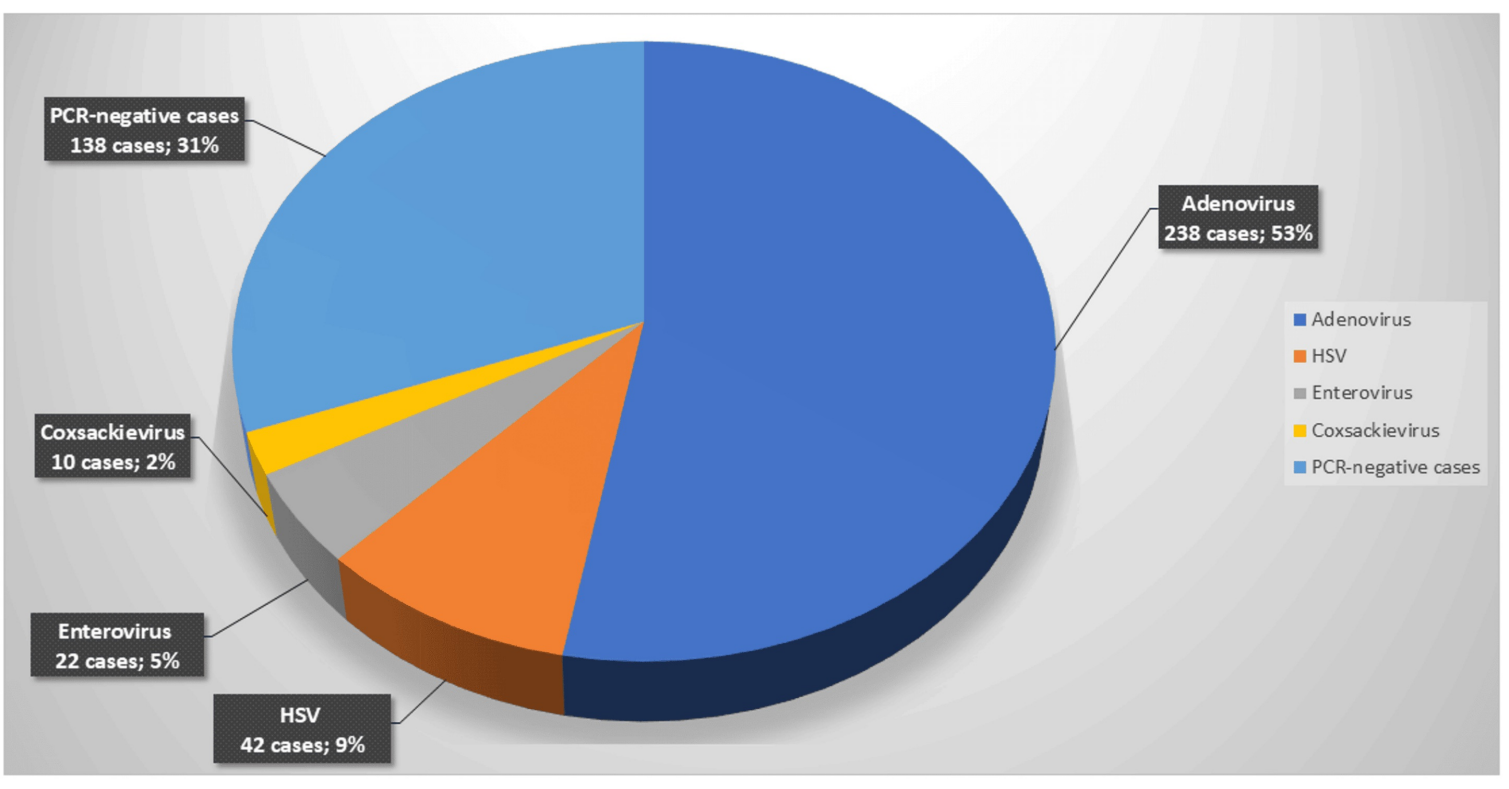
Prevalence of viral pathogens detected in conjunctival specimens.

Of the 450 samples, 312 (69.3%) tested positive for viral pathogens via real-time PCR. Adenovirus was the most prevalent, detected in 238 samples (52.9% of the total; 76.3% of viral positives), followed by HSV in 42 samples (9.3% of the total; 13.5% of viral positives), enterovirus in 22 samples (4.9% of the total; 7.1% of viral positives), and coxsackievirus in 10 samples (2.2% of the total; 3.2% of viral positives). A number of viral co-infections were identified. The remaining 138 samples (30.7%) tested negative for the targeted viruses, potentially indicating bacterial, allergic, or undetected viral etiologies, as shown in Figure 1.

Molecular Detection and Assay Performance

Real-time PCR assays demonstrated robust performance. The LOD was 10² copies/µL for adenovirus, HSV, and enterovirus, and 10³ copies/µL for coxsackievirus. Specificity was confirmed with no cross-reactivity against non-target pathogens. Duplicate runs on 45 samples (10%) showed 97.8% concordance, affirming reproducibility.

Viral Typing

Of the 238 adenovirus-positive samples, 210 (88.2%) were successfully typed using multiplex PCR targeting the hexon gene. The most common serotypes were Ad8 (n=82, 39.0%), Ad19 (n=54, 25.7%), Ad37 (n=36, 17.1%), Ad5 (n=22, 10.5%), and Ad11 (n=16, 7.6%). The remaining 28 samples (11.8%) were untypable, likely due to low viral load or novel variants. Among the 22 enterovirus-positive samples, CV-A24v (n=13, 59.0%), EV-70 (n=7, 31.8%), and Echo-11 (n=2, 9.1%) were identified, as shown in Table 2. Metagenomic deep sequencing of 20 selected samples (10 untypable and 10 typed for validation) confirmed multiplex PCR results in the typed samples and identified two untypable samples as Ad13, a serotype linked to severe outbreaks in India. Sequencing also detected trace HSV-2 in one sample, which was missed by initial PCR, suggesting rare subtype involvement.

**Table 2:** Genotypic distribution of major viral pathogens.

Virus	Genotype	n (%)	Regional predominance	Clinical association
Adenovirus	AdV-D8	82 (39.0%)	North (58.1%)	Severe follicular conjunctivitis
	AdV-D37	36 (17.1%)	South (42.3%)	Mild to moderate cases
	AdV-D19	54 (25.7%)	–	Epidemic outbreaks
	AdV-D5	22 (10.5%)	West (15.8%)	Prolonged symptoms
	AdV-D11	16 (7.6%)	–	Epidemic outbreaks
Enterovirus	CV-A24v	13 (59.0%)	Nationwide	Hemorrhagic conjunctivitis
	EV-70	7 (31.8%)	South (38.5%)	Rapid spread
	Echo-11	2 (9.1%)	–	Atypical presentation

Statistical analysis

Logistic regression analysis identified significant predictors of viral conjunctivitis: Contact history (OR = 2.18, 95% CI: 1.42-3.35, p < 0.001); Healthcare worker occupation (OR = 1.92, 95% CI: 1.05-3.51, p = 0.034); and Monsoon season (OR = 1.78, 95% CI: 1.12-2.83, p = 0.015). All values were adjusted for age and sex. Age and urban residence were not significant predictors in the multivariate model (p > 0.05).

Key observations

Adenovirus serotypes Ad8 and Ad19, which are associated with EKC, accounted for 64.7% of typed cases. These serotypes correlated with higher symptom severity, including photophobia and corneal involvement, reported in 68% (n = 90/132) of these cases. Prevalence peaked in younger adults and high-contact occupations, reflecting transmission dynamics in India’s densely populated urban centers and monsoon-related humidity that favors viral spread.

## Discussion

This study, conducted at SVBCH Hospital, Silvassa, provides a comprehensive analysis of the epidemiology, prevalence, molecular detection, and typing of viral pathogens in 450 conjunctival specimens collected from patients with acute conjunctivitis in India between January 2024 and December 2024. The findings reveal a high prevalence of viral etiologies (69.3%), with adenovirus dominating at 52.9%, followed by HSV, enterovirus, and coxsackievirus. These results align with global trends while offering unique insights into the Indian context, particularly regarding seasonal patterns, occupational risks, and serotype distribution. The observed viral prevalence of 69.3% is consistent with prior studies indicating that viral conjunctivitis accounts for a significant proportion of acute cases, often ranging from 65% to 80% globally [1,11]. Adenovirus, detected in 52.9% of samples, reaffirms its role as the predominant ocular pathogen, corroborating findings by Azari and Barney, who reported adenoviral conjunctivitis comprising up to 90% of viral cases [1]. The higher detection rate in this study compared to some regional reports (e.g., 36% in a South Indian outbreak [12]) may reflect the use of sensitive molecular techniques such as real-time PCR, which outperform traditional culture methods [13]. The presence of HSV (9.3%), enterovirus (4.9%), and coxsackievirus (2.2%) highlights a broader viral spectrum than is often reported, suggesting these pathogens may be underdiagnosed in India due to limited routine molecular testing [14]. The age-specific prevalence, peaking at 73.8% in the 19-40 age group, aligns with epidemiological patterns in which viral conjunctivitis predominates in adults, contrasting with the higher incidence of bacterial conjunctivitis in children [15]. This may reflect increased exposure in workplaces or educational institutions, a hypothesis supported by the significant association with occupations such as healthcare workers (80.0%) and students (75.0%). The strong link with contact history (78.5% positivity; OR = 2.18, p < 0.001) underscores the contagious nature of viral conjunctivitis, consistent with outbreak studies in India reporting household secondary attack rates as high as 47% [16]. These findings emphasize the need for targeted infection control measures in high-risk settings, such as hospitals and schools. Seasonal variation, with a peak during the monsoon (78.6%), is notable and likely tied to India’s humid climate facilitating viral transmission, as suggested by prior research linking adenovirus outbreaks to warm, wet conditions [17]. This contrasts with Western studies that report winter peaks [18], highlighting the influence of regional climate on epidemiology. The lack of a significant urban-rural disparity (71.3% vs. 66.1%, p = 0.227) differs from expectations of higher urban prevalence due to population density, possibly indicating widespread exposure across India’s diverse settings. The use of real-time PCR and metagenomic sequencing proved highly effective, detecting viruses in 69.3% of cases with a LOD of 10²-10³ copies/µL and 97.8% reproducibility. These results align with findings by Doan et al., where deep sequencing identified pathogens in 86% of conjunctival swabs, outperforming conventional diagnostics [19]. Adenovirus typing revealed Ad8 (39.0%) and Ad19 (25.7%) as dominant serotypes, both linked to EKC [20]. This mirrors a Tamil Nadu outbreak study that identified Ad8 and Ad19 among severe cases [21], suggesting these serotypes drive significant morbidity in India. The detection of Ad13 via sequencing in untypable samples further indicates the emergence of novel strains, warranting the expansion of typing panels in future studies. The absence of viral co-infections contrasts with some reports of mixed viral-bacterial infections, possibly due to the acute nature of cases sampled or the specificity of the assays used. However, the detection of HSV-2 traces via sequencing suggests rare subtypes may be missed by standard PCR, supporting the value of unbiased sequencing in comprehensive pathogen profiling. The high prevalence of EKC-associated serotypes (Ad8 and Ad19; 64.7% of typed adenoviruses), correlating with severe symptoms (68% of cases presenting with photophobia or corneal involvement), underscores the clinical burden of viral conjunctivitis in India. Misdiagnosis often leads to unnecessary antibiotic use, a concern highlighted by Yeu and Hauswirth. In the context of India’s antibiotic resistance crisis, accurate molecular diagnosis could help reduce inappropriate prescriptions, aligning with global estimates of $430 million in potential savings. The occupational and seasonal patterns observed in this study suggest that targeted interventions, such as hygiene campaigns in healthcare and educational settings during the monsoon, could curb transmission. Though the economic burden was not quantified here, it likely mirrors international figures (estimated at $377-857 million annually in the U.S.), emphasizing the need for cost-effective diagnostics, such as rapid assays (e.g., AdenoPlus), alongside PCR.

Limitations

Limitations of this study include its single-center design, which may affect generalizability across India’s diverse regions, and the exclusion of chronic or previously treated cases, which could underestimate prevalence. Additionally, the absence of viral load quantification (e.g., Ct values) and clinical severity correlation for typed serotypes limits the ability to link specific strains to clinical outcomes. The 30.7% (n = 138) of virus-negative samples likely reflect bacterial, allergic, or undetected viral etiologies, highlighting the need for broader diagnostic panels (e.g., including varicella-zoster virus).

Future studies should incorporate multicenter datasets, longitudinal outbreak tracking, vaccine development based on prevalent serotypes, and cost-effectiveness analyses of molecular diagnostics in India’s resource-limited settings.

## Conclusions

This study highlights the significant burden of viral conjunctivitis in India, primarily driven by adenovirus serotypes Ad8 and Ad19, with distinct epidemiological patterns linked to age, occupation, and the monsoon season. Molecular techniques provide a robust toolset for detection and typing, paving the way for improved diagnosis and effective outbreak management. These findings support the integration of advanced diagnostics into routine clinical practice to enhance patient care and strengthen public health responses in India.

## Appendices

Appendix 1 

**Table 3:** Supplementary dataset for metagenomic deep sequencing of conjunctival specimens.

Sample ID	Age	Gender	Season	Viral detection	Virus identified	Virus type	Accession number
CS001	14	Male	Monsoon	Positive	Adenovirus	Type 8	PP123456
CS002	45	Female	Non-monsoon	Negative	None	N/A	N/A
CS003	65	Male	Monsoon	Positive	Herpes simplex virus	HSV-1	PP123457
CS004	32	Female	Non-monsoon	Positive	Adenovirus	Type 3	PP123458
CS005	8	Female	Monsoon	Negative	None	N/A	N/A
CS006	50	Male	Non-monsoon	Negative	None	N/A	N/A
CS007	28	Female	Monsoon	Positive	Adenovirus	Type 4	PP123460
CS008	70	Male	Non-monsoon	Negative	None	N/A	N/A
CS009	19	Female	Monsoon	Positive	Adenovirus	Type 8	PP123461
CS010	55	Male	Non-monsoon	Positive	Herpes simplex virus	HSV-2	PP123462
CS011	12	Male	Monsoon	Negative	None	N/A	N/A
CS012	38	Female	Non-monsoon	Positive	Adenovirus	Type 7	PP123463
CS013	60	Male	Monsoon	Positive	Coronavirus	HCoV-HKU1	PP123464
CS014	25	Female	Non-monsoon	Negative	None	N/A	N/A
CS015	16	Female	Monsoon	Positive	Enterovirus	EV-B	PP123465
CS016	42	Male	Non-monsoon	Negative	None	N/A	N/A
CS017	33	Female	Monsoon	Negative	None	N/A	N/A
CS018	48	Male	Non-monsoon	Positive	Adenovirus	Type 4	PP123467
CS019	9	Female	Monsoon	Negative	None	N/A	N/A
CS020	52	Male	Non-monsoon	Negative	None	N/A	N/A
